# Grafting promoted antioxidant capacity and carbon and nitrogen metabolism of bitter gourd seedlings under heat stress

**DOI:** 10.3389/fpls.2022.1074889

**Published:** 2022-12-15

**Authors:** Le Liang, Wen Tang, Huashan Lian, Bo Sun, Zhi Huang, Guochao Sun, Xiaomei Li, Lihua Tu, Huanxiu Li, Yi Tang

**Affiliations:** ^1^ College of Horticulture, Sichuan Agricultural University, Chengdu, Sichuan, China; ^2^ Horticulture Research Institute, Chengdu Agricultural College, Chengdu, Sichuan, China; ^3^ Vegetable Germplasm Innovation and Variety Improvement Key Laboratory of Sichuan, Sichuan Academy of Agricultural Sciences, Chengdu, Sichuan, China; ^4^ College of Forestry, Sichuan Agricultural University, Chengdu, Sichuan, China; ^5^ Institute of Pomology and Olericulture, Sichuan Agricultural University, Chengdu, Sichuan, China

**Keywords:** carbohydrate, correlation analysis, luffa, nitrogen, pumpkin, PCA

## Abstract

**Introduction:**

Heat stress can limit vegetable growth, and this can lead to constraints on agricultural production. Grafting technologies, however, can be used to alleviate various plant stresses.

**Methods:**

In this study, the differences in the heat stress impacts and recovery abilities of pumpkin and luffa rootstocks for bitter gourd were analyzed in terms of their antioxidant activity and carbon and nitrogen metabolism.

**Results:**

Compared with the un-grafted and self-grafted bitter gourd, which suffered from heat stress at 40°C for 24 h, heterologously grafted bitter gourd showed higher heat stability of the cell membrane (relative conductivity and malondialdehyde content were reduced), reduced oxidative stress (antioxidant enzyme activity was increased and the reactive oxygen species content reduced), and increased enzyme activity (sucrose phosphate synthase, sucrose synthase, neutral invertase, and acid invertase) and sugar content (soluble sugar, sucrose, fructose, and glucose) in carbon metabolism. The enzyme activity (nitrate reductase, nitrite reductase, and glutamine synthetase) and product content (nitrate and nitrite) of nitrogen metabolism were also found to be increased, and this inhibited the accumulation of ammonium ions. After the seedlings were placed at 25°C for 24 h, the heterogeneous rootstocks could rapidly restore the growth of the bitter gourd seedlings by promoting the antioxidant and carbon and nitrogen metabolism systems. When luffa was used as rootstock, its performance on the indexes was better than that of pumpkin. The correlation between the various indicators was demonstrated using a principal component and correlation analysis.

**Discussion:**

The luffa rootstock was found to be more conducive to reducing cell damage and energy loss in bitter gourd seedlings caused by heat induction through the maintenance of intracellular redox homeostasis and the promotion of carbon and nitrogen metabolism.

## 1 Introduction

To ensure that there is an annual supply of various vegetables, autumn-delayed cultivation of protected plots has been introduced in different regions. This stubble arrangement of vegetable cultivation has been found to satisfy consumer needs. However, high temperatures in the autumn can lead to stunted growth and even death for some vegetables, which seriously affects their economic benefits. In the process of high temperature, plant cells will produce oxygen free radicals and their derivatives, namely reactive oxygen species (ROS), and make the balance between ROS generation and antioxidant defense system imbalance, thus triggering the excessive accumulation of ROS and inducing oxidative stress in plants (Hasanuzzaman et al., 2020a). It is worth noting that both enzymatic and non-enzymatic antioxidant defense systems can maintain the balance between detoxification and production of ROS. Among them, the endogenous defense mechanisms composed of different enzymes include: superoxide dismutase, SOD; catalase, CAT; peroxidase, POD; ascorbic acid peroxidase, APX; enzyme in the glutathione ascorbic acid cycle, AsA-GSH. Non-enzymatic antioxidants include ascorbic acid, AsA; glutathione, GSH; flavonoids, carotenoids, etc (Hasanuzzaman et al., 2020b). Under heat stress, the solubility of CO_2_ and O_2_ in plant leaf cells decreases, which consequently means that the amount of CO_2_ fixed by ribulose-1,5-diphosphate carboxylation/oxygenase decreases, while the oxidation reaction in photorespiration and the CO_2_ diffusion resistance increases, and these conditions are not conducive to leaf photosynthesis (Ara et al., 2013). Under 35 °C heat stress, the cell membranes in the leaves of cucumber (*Cucumis sativus* L.) seedlings were damaged due to the excessive accumulation of soluble protein, proline (Pro), malondialdehyde (MDA), and superoxide anion ( ${\text{O}}_{2}^{•-}$ ) (Ding et al., 2016). This resulted in reduced cell membrane fluidity and damaged the DNA, proteins, and lipids of the plant (Giordano et al., 2021). This also stimulates the activity of antioxidant enzymes and the expression of related genes such as glutathione reductase, SOD, POD, and CAT (Ding et al., 2016).

Carbon and nitrogen metabolism are two irreplaceable processes in plant growth and development but are affected by heat stress. Carbohydrates play a positive role in plant growth, photosynthesis, carbon partitioning, lipid metabolism, osmotic homeostasis, protein synthesis, and gene expression under heat stress (Rosa et al., 2009; Sami et al., 2016), as they can help maintain the stability of the cell membrane (Lemoine, 2013). Heat stress can cause soluble sugars, fructose, and sucrose to accumulate in plant leaves (Yuan et al., 2014; Shan et al., 2016). Carbohydrates play a key role in osmoregulation and protect cell membranes from dehydration (Ruan et al., 2010), and increasing the carbohydrate content and the activity of sucrose-degrading enzymes can alleviate the physiological damage to leaves under heat stress (Loka et al., 2020). Moreover, leaf carbohydrate content is largely dependent on sucrose metabolism. Heat stress (40 °C) increased the sucrose content in melons (*Cucumis melo* L. cv. Hale’s Best Jumbo), and this may be due to the upregulated expression of the sucrose transporter (Gil et al., 2012). Nitrogen metabolism in plants is closely related to photosynthesis and respiration, and plants can absorb inorganic nitrogen ( ${\text{NO}}_{3}^{-}$ and ${\text{NH}}_{4}^{+}$ ) from the soil. After ${\text{NO}}_{3}^{-}$ enters the cell, it is reduced to ${\text{NO}}_{2}^{-}$ by nitrate reductase (NR) and ${\text{NO}}_{2}^{-}$ is reduced to ${\text{NH}}_{4}^{+}$ by nitrite reductase (NiR). ${\text{NH}}_{4}^{+}$ is then assimilated into amino acids and other organic matter through the glutamine-glutamate cycle pathway (Liang et al., 2021), which requires energy for metabolism. However, various environmental stressors can lead to insufficient energy metabolism in plants, which further affects their absorption of inorganic nitrogen. When subjected to heat stress, the activities of NR, glutamine synthase (GS), glutamate synthase (GOGAT), and glutamate dehydrogenase (GDH) in the leaves of heat-tolerant plants were found to be higher, but the total amino acid level in the leaves did not change (Yuan et al., 2017).

There are numerous ways to alleviate the damage caused by heat stress in plants. Grafting is a type of vegetative asexual reproduction, and it utilizes plant totipotency to a plant branch or bud cambium and another plant stem or root cambium so that the two parts grow into a complete plant. The ability of plants to resist abiotic stress can be improved by grafting, mainly due to the resistance of the rootstock. Heat-resistant rootstocks have been shown to have strong growth and high ROS scavenging activity under heat stress conditions, and the glutathione and ascorbic acid contents in the seedlings were also found to be high (Zhang et al., 2007). When luffa was used as a rootstock, CO_2_ assimilation and the related enzyme activities of cucumber grafted seedlings were promoted (Li et al., 2014a). Under heat stress (40 °C), the leaves of the cucumber seedlings and self-grafted seedlings accumulated more ROS when luffa was used as the rootstock, the increase in hydrogen peroxide (H_2_O_2_) and ${\text{O}}_{2}^{•-}$ in the grafted seedlings was alleviated, and the activities of the antioxidant enzymes were increased, which improved the heat tolerance of the cucumber seedlings (Li et al., 2016) Li et al. have obtained similar results (Li et al., 2014b). However, the effects of grafting on carbon and nitrogen metabolism in melon vegetables have not been widely investigated.

Bitter gourd (*Momordica charantia* L.) is an annual climbing tender herb native to eastern India. It is widely planted worldwide, from tropical to temperate zones, and in particular, in the southern and northern areas of China. In the actual production process, bitter gourd seedlings will be exposed to excessive heat stress in the late autumn cultivation. Bitter gourd has been used as a rootstock with strong stress resistance in numerous studies. Using bitter gourd as a rootstock can improve the heat resistance (40 °C) of cucumber seedlings. Proteome analysis can increase the protein content involved in energy metabolism, defense responses, and protein and nucleic acid biosynthesis (Xu et al., 2018). Wei et al. (2019) found that cucumber plants grafted with bitter gourd could maintain the homeostasis of redox in cells after heat stress (40 °C). However, there are few reports on improving the heat resistance of bitter gourds using other resistant rootstocks. In this study, we analyzed the effects of different rootstocks on the heat tolerance of bitter gourd in terms of antioxidant activity and carbon and nitrogen metabolism levels and identified grafting mechanisms that could be utilized to improve stress resistance in bitter gourd.

## 2 Materials and methods

### 2.1 Materials

The experiment was conducted at the Chengdu Campus of the Sichuan Agricultural University from August to November 2021. The scion used was a bitter gourd (*Momordica charantia* L.), F-1437, hereafter referred to as M. Two types of rootstock were also utilized: pumpkin (*Cucurbita moschata* (Duch. ex Lam.) Duch. ex Poiret), hereafter referred to as C; and luffa (*Luffa cylindrica* L.), hereafter referred to as L. The stock and scion materials were obtained from the Chengdu Agricultural Vocational and Technical College.

### 2.2 Experimental design

The seeds of grafted rootstocks and scions were soaked in 45–55°C water for 30 minutes and were then soaked for 24 h after cooling. After the seeds expanded, they were put into a petri dish containing wet filter paper to accelerate germination at 30 °C. When the white tip of the seeds reached 0.5 cm, they were sown. The stock was sown 2-3 days before the scion (perlite: vermiculite = 2:1). When the rootstock had one leaf and one heart and the first true leaf of the scion was unfolded, split grafting was performed. There were four grafting treatments: bitter gourd seedlings (M), self-grafted bitter gourd seedlings (MS), pumpkin as the rootstock and bitter gourd as the scion (MC), and luffa as the rootstock and bitter gourd as the scion (ML).

While grafting, a transparent plastic film was used to cover the grafts. After grafting, a black film was used to cover for shading. The cover must be placed for the whole day for shading, heat preservation, and moisture preservation over 1-3 days. Within 4-6 days after grafting, light was blocked from 10 a.m. to 3 p.m. every day, and the black film was removed for the rest of the time. The temperature in the small arch shed was controlled at 32 °C during the day and 20 °C at night, and the humidity was controlled at ~90%. After 7 days, the plants were placed under light all day, and the seedlings were gradually ventilated and cooled to keep the temperature at 25 °C during the day and 18 °C at night. After 10-15 days of grafting, the grafting clip was removed.

When the grafted seedlings had grown four leaves and one heart, seedlings were selected with uniform growth vigor and placed in an intelligent artificial incubator (Tianjin Taist Instrument Co., Ltd., RGX300EF) for pre-culture. There were 20 pots per treatment, 1 plant per pot, and 3 replicates per treatment. The pre-culture conditions were as follows: 25/18 °C, 12 h/12 h (day/night), light intensity 300 μmol·m^-2^·s^-1^, and relative humidity 80%. After 24 h of pre-culture, heat stress (H) was performed: 40 °C, 12/12 h (day/night), the light intensity was 300 μmol·m^-2^·s^-1^, and the relative humidity was set to 80%. After 24 h of heat treatment, all treated seedlings were placed in an incubator at 25 °C under a 12 h light/12 h dark cycle, the light intensity was 300 μmol·m^-2^·s^-1^, and the relative humidity was 80% for 24 h, to restore the growth of seedlings after heat stress (RH). The grafted seedlings were randomly arranged in an intelligent artificial incubator, and their positions were changed regularly to ensure consistent growth conditions and adequate moisture. Samples were collected and measured 24 h after heat stress (H) and 24 h after the recovery of growth (RH).

### 2.3 Determination of indices

#### 2.3.1 Relative conductivity and osmotic regulating substances

The relative conductivity of the leaves was measured using a conductance instrument (Shanghai INESA Scientific Instrument Co., Ltd, DDS-307). Soluble protein content was determined using the Coomassie brilliant blue G-250 method, MDA content was determined using the thiobarbituric acid method, and Pro content was determined using the acid ninhydrin colorimetric method (Wang and Huang, 2015). The ${\text{O}}_{2}^{•-}$ content was determined using the p-aminobenzenesulfonic acid method (Ke et al., 2007) and the H_2_O_2_ content was determined using potassium iodide spectrophotometry (Chakrabarty and Datta, 2008).

#### 2.3.2 Antioxidant enzyme activity

In preparation for the antioxidant enzyme extract, leaves (0.5 g) were weighed and ground with 5 mL of 50 mmol·L^-1^ phosphate buffer (pH 7.8) containing 0.2 mmol·L^-1^ EDTA and 2% polyvinylpyrrolidone. The homogenate was centrifuged at 12000 × *g* for 20 min at 4 °C, and the supernatant was then used for the determination of antioxidant enzyme activity. SOD activity was measured using the nitroblue tetrazolium method, POD activity using the guaiacol method, and CAT activity using the ultraviolet (UV) absorption method (Wang and Huang, 2015).

#### 2.3.3 Carbon metabolism and related enzyme activities

The total soluble sugar content was determined using the anthrone method (Wang and Huang, 2015), sucrose and fructose were determined using the Buyssehe and Merckx’s method (1993), and glucose content was determined using a colorimetric method (Jones et al., 1977).

The enzyme solution was prepared according to the method of Holaday et al. (1992), and the activity of the sucrose phosphate synthase (SPS) was measured. To measure the activities of sucrose synthase (SS), neutral synthase (NI), and acid synthase (AI), enzyme solutions were prepared as previously described (Shahid et al., 2018).

#### 2.3.4 Nitrogen metabolism and related enzyme activities

The free amino acid content was determined using the ninhydrin solution chromogenic method (Wang and Huang, 2015). The ${\text{NO}}_{3}^{-}$ and ${\text{NO}}_{2}^{-}$ contents were determined according to Cataldo et al. (2008). The ${\text{NH}}_{4}^{+}$ content was determined according to the method described by Molins-Legua et al. (2006).

NR activity was measured using an *in vitro* method (Wang and Huang, 2015), NiR activity was measured according to the method described by Ramarao et al. (1983), and GS was measured according to the method of Lillo (1984).

### 2.4 Statistical analysis

All data were sorted using Excel 2010 software. Statistical analyses were conducted using SPSS 20.0 (IBM Corporation, Armonk, NY, USA). Data were analyzed using one-way analysis of variance, followed by Duncan’s new complex range method at a 5% level of significance (*P*< 0.05). In order to study the relationship between various indicators, we conducted principal component analysis (PCA) and correlation analysis.

## 3 Results

### 3.1 Changes in the ROS and osmotic substance levels

**Figure 1:**
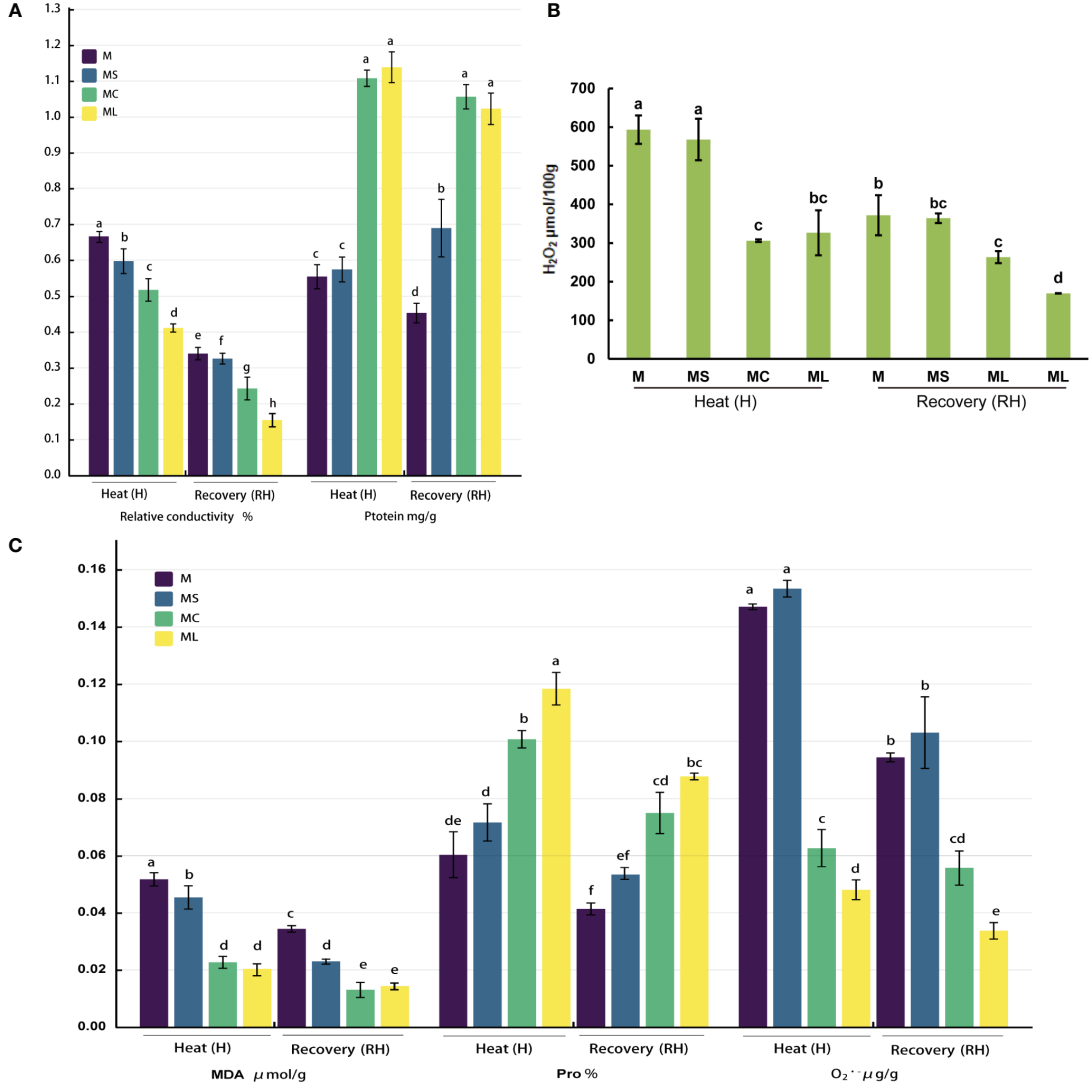
Changes in the ROS and osmotic substance levels. **(A)** Relative conductivity and protein content. **(B)** H_2_O_2_ content. **(C)** MAD, Pro and ${\text{O}}_{2}^{•-}$ content. Values are the mean (± SE) of three replicates. Different letters indicate statistically significant differences between the treatment groups (*P<*0.05).

After heat stress or recovery, the relative conductivity and MDA, ${\text{O}}_{2}^{•-}$ , and H_2_O_2_ contents of the MC and ML decreased significantly when compared with the M and MS; at the same time, the protein and Pro contents increased significantly (**Figure 1**). In addition, when comparing ML with MC, regardless of heat stress or recovery, the relative conductivity and ${\text{O}}_{2}^{•-}$ content in the ML was lower than that of the MC (under heat stress, it decreased by 20.47% and 23.72% respectively, and decreased by 36.48% and 39.82% respectively after recovery), while the protein and MDA of the ML were not significantly different from those of the MC. After heat stress, the Pro content in the ML was significantly higher (17.36%) than that of the MC, but after recovery, there was no significant difference between the two. The results for the H_2_O_2_ content levels were the opposite. When MS and M were compared, the contents of Pro, ${\text{O}}_{2}^{•-}$ , and H_2_O_2_ did not differ significantly with the heat stress or after recovery, whereas the relative conductivity and MDA content of the MS were both significantly lower than those of the M. After the heat stress, the protein content of the MS was not significantly different from that of the M; however, after recovery, the protein content of the MS was significantly higher than that of the M.

### 3.2 Activity of antioxidant enzymes

**Figure 2:**
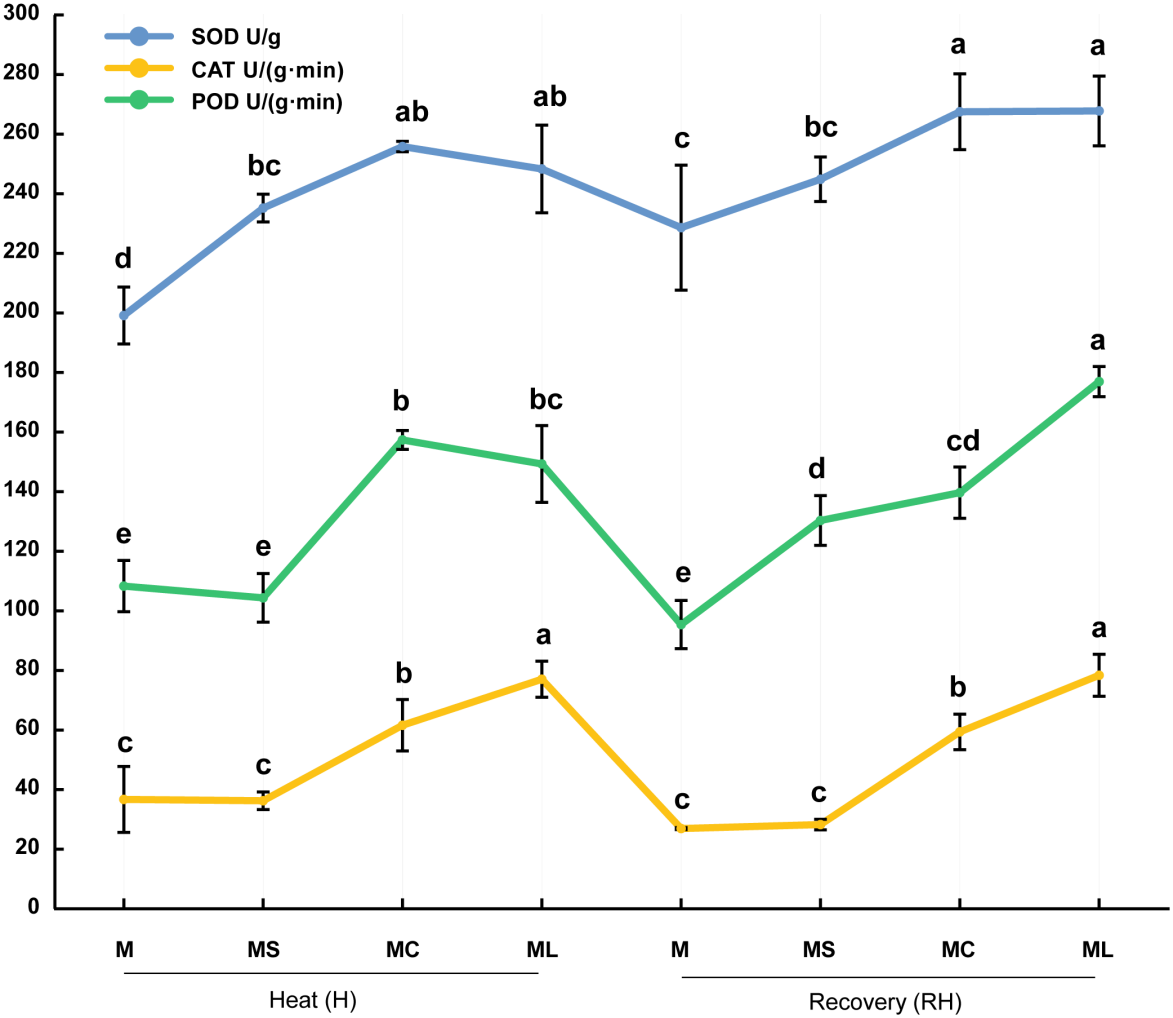
Activity of antioxidant enzymes. Values are the mean (± SE) of three replicates. Different letters indicate statistically significant differences between the treatment groups (*P<*0.05).

When bitter gourd seedlings were subjected to high-temperature stress or resumed growth at 25 °C, the activities of SOD, POD, and CAT in the MC and ML were significantly higher than those in the M and MS (**Figure 2**). At the same time, after heat stress or recovery, the CAT activity of the ML was significantly higher than that of the MC (increased by 25.18% and 32.14% respectively), whereas the SOD activity of the ML was not significantly different from that of the MC. In terms of POD activity, after the heat stress, there was no significant difference between the ML and MC; however, after recovery, the ML was significantly higher (26.73%) than that of the MC. After heat stress, the activity of the POD and CAT in the MS was not significantly different from that of the M, but the activity of the SOD was significantly increased (18.12%). After recovery, the activities of SOD and CAT in the MS were not significantly different from those in the M, but the activity of the POD was significantly increased.

### 3.3 Carbon metabolite content

**Figure 3:**
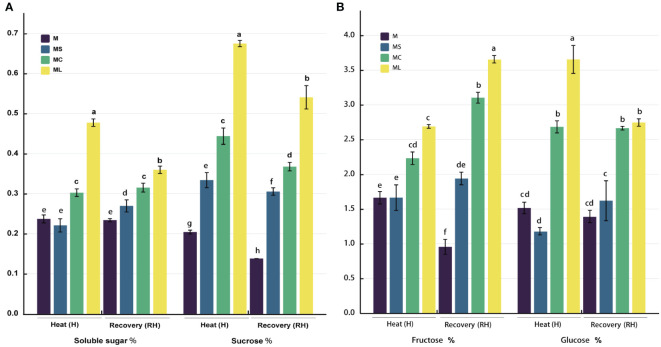
Carbon metabolite content. **(A)** Soluble sugar and sucrose content. **(B)** Fructose and glucose content. Values are the mean (± SE) of three replicates. Different letters indicate statistically significant differences between the treatment groups (*P<*0.05).

After heat stress or growth recovery, the soluble sugar, sucrose, fructose, and glucose contents in the ML and MC were significantly higher than those in the M and MS, and the soluble sugar and sucrose contents in the ML were significantly higher than those in the MC, the soluble sugar content increased by 57.74% and 14.27%, and the sucrose content increased by 52.01% and 42.94%, respectively (**Figure 3**). After heat stress, the fructose content of the ML was not significantly different from that of the MC; however, the fructose content of the ML was significantly higher (17.70%) than that of the MC after growth recovery. After heat stress, the glucose content in the ML and MC increased significantly (36.24%), but there was no significant difference between ML and MC after growth recovery. After heat stress, the soluble sugar, fructose, and glucose contents of the MS were not significantly different to those of M, but the sucrose content of MS was significantly higher than that of M. After recovery at 25 °C, the soluble sugar, sucrose, and fructose contents in MS were significantly higher than those in M, but there was no significant difference in glucose.

### 3.4 Activity of carbon metabolism enzymes

**Figure 4:**
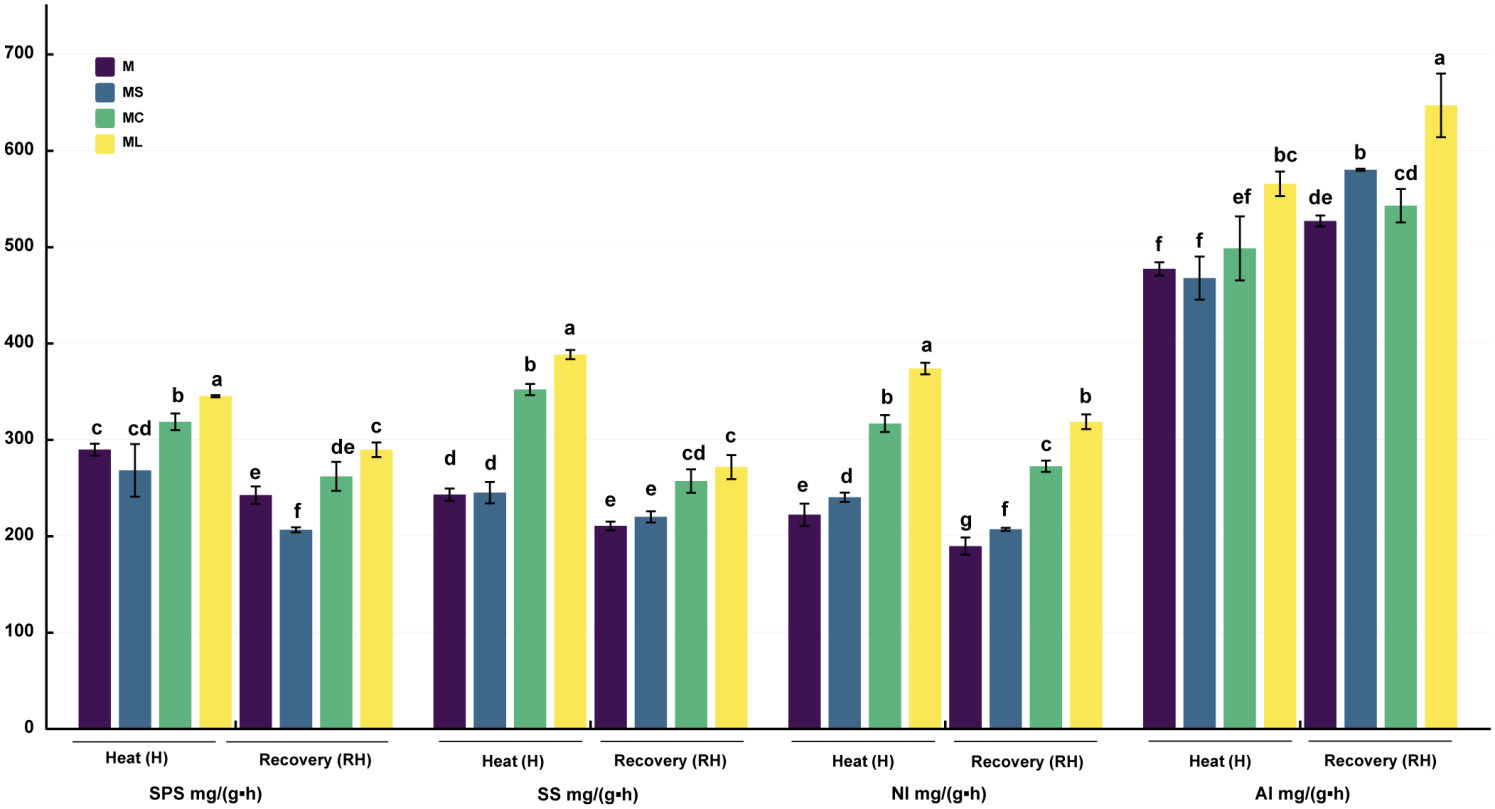
Activity of carbon metabolism enzymes. Values are the mean (± SE) of three replicates. Different letters indicate statistically significant differences between the treatment groups (*P<*0.05).

The SPS, SS, and NI activities of MC and ML were significantly higher than those of M and MS after heat stress and recovery, and the activities of ML were significantly higher than those of MC, except that the SS (RH) activities of ML and MC were not significantly different (**Figure 4**). Compared with MC, the SPS activity of ML significantly increased by 8.36% and 10.56% after heat stress and recovery, and the NI activity increased by 18.06% and 16.95% respectively, while the SS activity increased by 10.32% only after heat stress. After heat stress, the activities of SPS and SS in the MS were not significantly different from those in M; however, the activities of NI were significantly increased. After the recovery of growth, the activities of the SPS, SS, and NI of MS were significantly reduced, with no significant difference, and were significantly increased when compared with that of M. For AI activity, after the heat stress or recovery, ML was significantly higher than that for other treatments. After heat stress, the AI activity of ML was significantly increased by 18.25%, 12.67% and 16.37% compared with M, MS and MC respectively, and 22.79%, 11.53% and 19.18% respectively after the recovery. After heat stress, there was no significant difference in the AI activity among the M, MS, and MC groups. After recovery, the AI activity of MS was significantly higher than that of M, and there was no significant change in MC.

### 3.5 Nitrogen metabolite content

**Figure 5:**
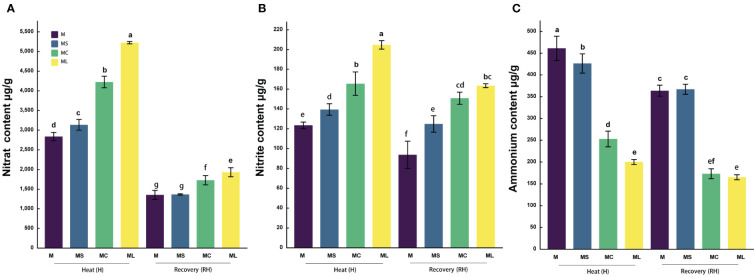
Nitrogen metabolite content. Values are the mean (± SE) of three replicates. Different letters indicate statistically significant differences between the treatment groups (*P<*0.05). **(A)** Nitrate content. **(B)** Nitrite content. **(C)** Ammonium content.

After the heat stress and recovery, the ${\text{NO}}_{3}^{-}$ and ${\text{NO}}_{2}^{-}$ contents of the grafted seedlings in MC and ML were significantly higher than those of the MS and M, and the ${\text{NH}}_{4}^{+}$ contents were significantly higher than those of MS and M (**Figure 5**). After heat stress, the ${\text{NO}}_{3}^{-}$ and ${\text{NH}}_{4}^{+}$ contents in the ML were significantly higher (23.6%) and lower (20.85%) than those in the MC, respectively, but there was no significant difference in ${\text{NO}}_{2}^{-}$ . After recovery, the ${\text{NO}}_{3}^{-}$ content in the ML was significantly higher (11.73%) than that in the MC, but the ${\text{NO}}_{2}^{-}$ and ${\text{NH}}_{4}^{+}$ contents were not significantly different from those in the MC. The ${\text{NO}}_{3}^{-}$ and ${\text{NO}}_{2}^{-}$ contents in the MS were significantly higher than those in the M after heat stress, 10.55% and 12.94% respectively, while the ${\text{NH}}_{4}^{+}$ content was significantly reduced by 7.59%. After recovery, the ${\text{NO}}_{3}^{-}$ and ${\text{NH}}_{4}^{+}$ contents in the MS were not significantly different from those in the M, but the ${\text{NO}}_{2}^{-}$ content increased significantly.

### 3.6 Activity of nitrogen metabolism enzymes

**Figure 6:**
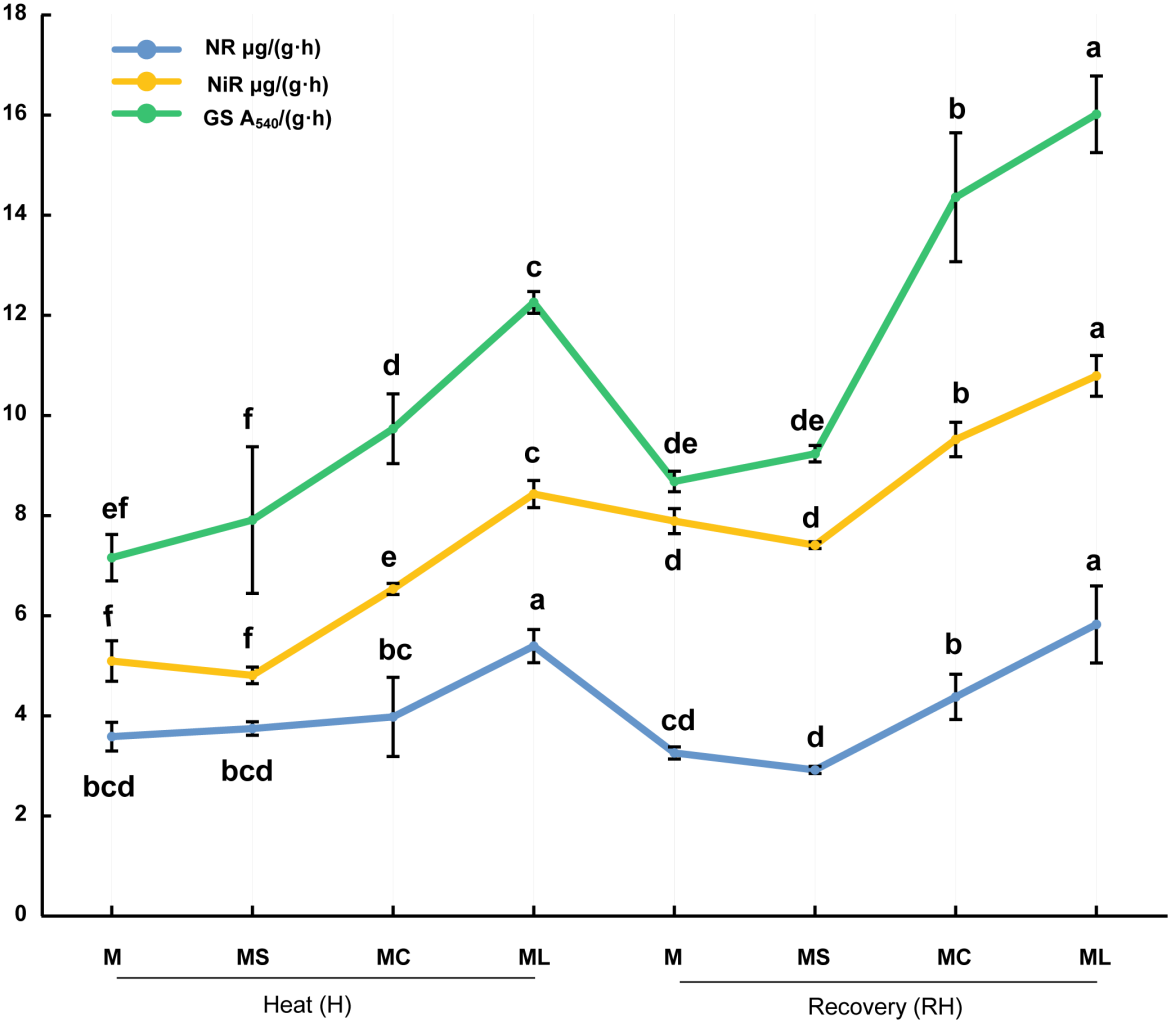
Activity of nitrogen metabolism enzymes. Values are the mean (± SE) of three replicates. Different letters indicate statistically significant differences between the treatment groups (*P<*0.05).

After heat stress and recovery, NR, NiR, and GS activities in the MC and ML of the grafted seedlings were significantly higher than those in the MS and M (**Figure 6**). Except after heat stress, there was no significant difference in NR activity between the MC, M, and MS. At the same time, after heat stress and recovery, the NR, NiR, and GS activities of ML were significantly higher than those of the MC. Compared with MC, the activities of NR, NiR and GS in ML increased by 35.67%, 29.09% and 25.96% respectively after heat stress, and increased by 33.20%, 13.37% and 11.56% respectively after recovery. After heat stress and recovery, there were no significant differences in the NR, NiR, and GS activities between the MS and M.

### 3.7 Principal component and correlation analysis

**Figure 7:**
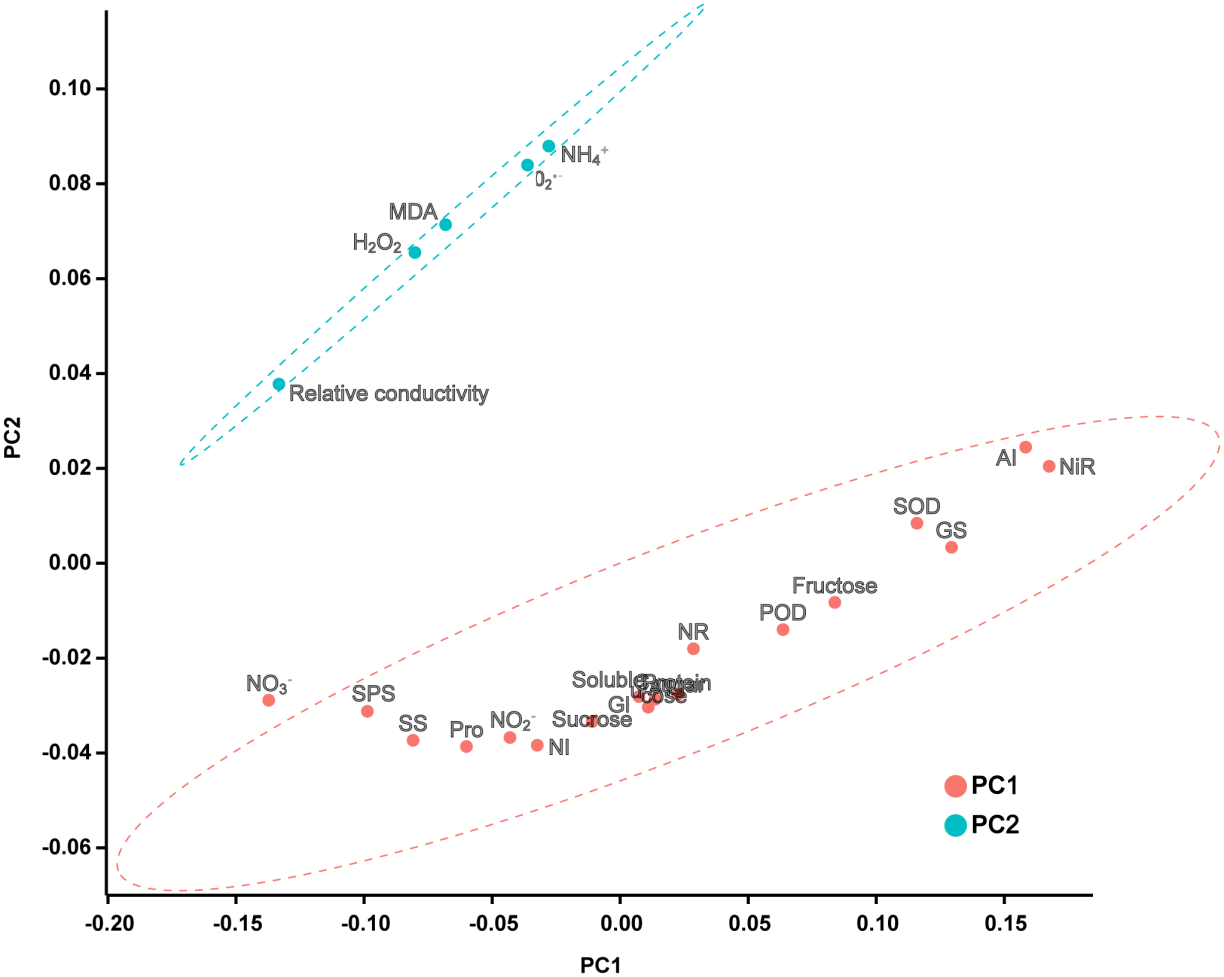
PCA score plot of each indicator. According to the principle that the eigenvalue is greater than 1, two principal components are extracted with a confidence interval of 95%.

Two principal components were extracted from the principal component analysis, and the characteristic root values were > 1 (**Figure 7**; **Supplementary Table 1**). The variance interpretation rates of the two principal components were 65.791% (PC1) and 21.459% (PC2), and the cumulative variance interpretation rate was 87.250%. Relative conductivity, MDA, ${\text{O}}_{2}^{•-}$ , H_2_O_2_, and ${\text{NH}}_{4}^{+}$ were negatively correlated with PC1. The 23 indicators were divided into two groups: one group was composed of cell damage indicators and the other group was composed of antioxidants and carbon and nitrogen metabolism systems.

**Figure 8:**
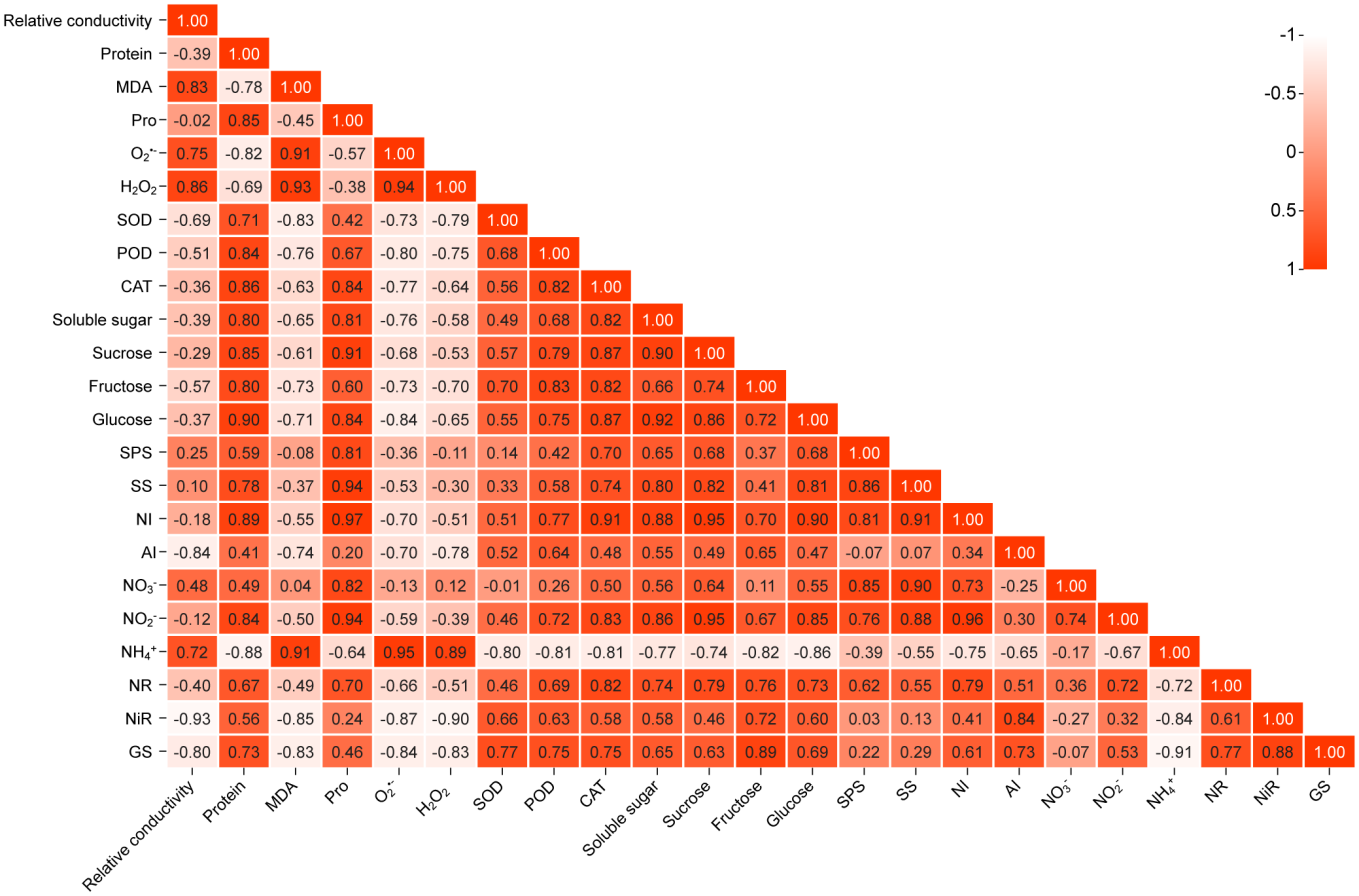
Correlation diagram of correlation of three indicators. Positive values indicate positive correlation, while negative values indicate negative correlation. The darker or lighter the color, the stronger the correlation between the indicators. All correlations in the figure reflect the absolute value of the Pearson correlation coefficient above the threshold (*P<*0.05, *P<*0.01).

To further intuitively display the relationship between the various indicators, a correlation analysis of the data was conducted based on the heat map, as shown in **Figure 8**. The correlation coefficient values between relative conductivity and the 11 items of MDA, ${\text{O}}_{2}^{•-}$ , H_2_O_2_, SOD, POD, fructose, AI, ${\text{NO}}_{3}^{-}$ , ${\text{NH}}_{4}^{+}$ , NiR, and GS showed a significant difference (*P<*0.05, *P<*0.01). There was a significant positive correlation between relative conductivity and MDA, ${\text{O}}_{2}^{•-}$ , H_2_O_2_, and ${\text{NH}}_{4}^{+}$ (correlation coefficients were 0.826, 0.750, 0.859, and 0.721, respectively; *P<*0.01). There was a significant negative correlation between the relative conductivity and SOD (-0.685, *P<*0.01), POD (-0.505, *P<*0.05), fructose (-0.572, *P<*0.01), AI (-0.840, *P<*0.01), ${\text{NO}}_{3}^{-}$ (0.483, *P<*0.05), NiR (-0.933, *P<*0.01), and GS (-0.802, *P<*0.01). The correlation value between relative conductivity and the 11 items of protein, Pro, CAT, soluble sugar, sucrose, glucose, SPS, SS, NI, ${\text{NO}}_{2}^{-}$ , and NR were not significant (*P* > 0.05), indicating that there was no correlation between relative conductivity and these 11 items.

## 4 Discussion

For optimal growth and development, vegetables often require an optimal temperature, and they can perceive slight changes in the ambient temperature, such as increases; these will greatly affect the physiological and metabolic processes of vegetables (Giordano et al., 2021). Photosynthesis is one of the processes most affected by temperature, and this can cause large disruptions to energy allocation and the final biomass production of vegetables. Previous studies have found that heat stress not only changes the structure of the thylakoid membrane and pigment protein complex (Rath et al., 2022), but also reduces leaf water potential (Weng et al., 2021). Exposure to stress temperatures leads to a decrease in CO_2_ assimilation, resulting in an increase in PSII electron transport, which in turn enables chloroplasts to accumulate large amounts of ROS (Zhou et al., 2007; Rath et al., 2022). Grafting is an ecologically sustainable agricultural practice that can improve stress resistance in vegetables. Moreover, studies have found that the effects of grafting depend on the characteristics of the scion and rootstock as well as their interactions (Colla et al., 2010). In a grafting experiment using cucumber, the rootstock was found to significantly increase the activities of the antioxidant enzymes (CAT and POD), improve the accumulation of Pro, and reduce lipid peroxidation (MDA) content (Shehata, 2022). Moreover, by improving the carboxylation efficiency, transcription of defense-related genes, and scavenging activity of ROS, the oxidative stress of grafted watermelon seedlings was significantly reduced (Li et al., 2014a). In our study, after heat stress, the two different rootstocks increased the Pro and protein content, decreased relative conductivity and MDA content, and inhibited ROS (H_2_O_2_ and ${\text{O}}_{2}^{•-}$ ) accumulation by promoting antioxidant enzyme (SOD, POD, and CAT) activities in the leaves of bitter gourd seedlings, when compared with the self-rooted grafted seedlings. After recovery at 25 °C, the grafted seedlings of the pumpkin and luffa were still superior to the self-grafted and non-grafted seedlings for all indicators. At the same time, under heat stress or after recovery, the luffa had a more significant effect on alleviating oxidative damage in bitter gourd seedlings. These results are similar to those reported by Shehata (2022) and Li et al. (2014b). Relative electrical conductivity and MDA represent cell integrity and membrane lipid peroxidation in leaves, and their reduced values indicate that seedlings have higher stress resistance and that cell damage can be prevented. Previous studies have shown that increased Pro content can reduce photo inhibition to maintain electron conduction between the two photosystems (Ashraf and Foolad, 2007; Szabados and Savouré, 2010; Padilla et al., 2021), which acts as a low molecular weight cellular antioxidant that removes free radicals and protects plants from oxidative damage (Anjum et al., 2012). During normal growth and development of plants, the production and removal of ROS in cells is balanced (Mittler, 2017), but the excessive accumulation of ROS not only destroys cellular components (carbohydrates, proteins, lipids, etc.), but also leads to cell death (Raja et al., 2017). Plants have enzymatic and non-enzymatic antioxidant defense systems; the enzymes in these systems mainly include SOD, POD, and CAT. SOD first converts ${\text{O}}_{2}^{•-}$ to H_2_O_2_ in response to high-temperature stress, and the generated H_2_O_2_ is further converted to H_2_O by CAT or POD (Biczak, 2016). Cucumber seedlings using bitter gourd (Tao et al., 2020), melon (Miao et al., 2019), or luffa (Li et al., 2016) as rootstocks all showed good antioxidant activity. Luffa is a type of gourd with strong heat tolerance, which is conducive to improving the oxidation resistance of scions. In plants, in addition to antioxidant enzymes, the role of low molecular weight antioxidants can’t be ignored. AsA has the potential to provide electrons as a coenzyme. It clears ROS by regulating the water state of cells (Hasanuzzaman et al., 2019). Tocopherol is mainly synthesized in the photosynthetic apparatus and protects the photosynthetic membrane by clearing ROS (Hasanuzzaman et al., 2014). Carotenoids are mainly responsible for pigment deposition and ROS quenching capacity. Flavonoids reduce plant cell damage by scavenging free radicals and protecting the cell membrane from membrane lipid peroxidation (Løvdal et al., 2010). Therefore, in the future research, more attention should be paid to the contribution of non-enzymatic antioxidants to abiotic stress resistance of plants.

In this study, heat stress increased the content of the total soluble sugar, sucrose, fructose, and glucose in grafted bitter gourd seedlings, especially in the two heterologous rootstocks. The accumulation of sugar in plant leaves depends on photosynthesis and the transport of carbohydrates, and the stress resistance of grafted plants can be achieved by significantly increasing the content of the total soluble sugar, reducing sugar, and sucrose (Turhan and Ergin, 2012). Grafted seedlings can degrade more starch, which is not only conducive to respiration and growth, but also to the maintenance of a high soluble sugar content (Sato et al., 2004), so that plants can accumulate more energy to resist stress. Interestingly, the contents of sucrose and fructose in the leaves of the self-grafted bitter gourd seedlings also increased correspondingly, which may be due to the transport of sugar and its accumulation at the grafting site or the formation of vascular bundles. This is similar to the results of Miao et al. (2019), in which it was suspected that the rapid accumulation of biomass in pumpkin/pumpkin compared to cucumber/cucumber and cucumber/pumpkin may be due to early vascular bundle reconnection. The bitter gourd seedlings grafted with luffa as rootstock had a higher sugar content to reduce the negative effects caused by heat stress. This may be because luffa roots have strong heat resistance (Zhang et al., 2007). The activities of AI, NI, SS, and SPS in plant leaves affect the contents of non-reducing sugars (sucrose) and reducing sugars (fructose and glucose). In this study, the contents of the non-reducing and reducing sugars were directly proportional to the related enzyme activities. In heterologous bitter gourd-grafted seedlings, the increase in leaf SPS activity promoted sucrose accumulation; SS and NI were conducive to the accumulation of glucose and fructose, while AI was inferior to the first three enzyme activities. This is similar to the results of Shahid et al. (2018), but in a study by Turhan and Ergin (2012), stress resistance was found to increase with the reduction of NI and SS activities in the bark tissue of 4-year-old sweet cherry-grafted trees. This phenomenon largely depends on where the samples are taken from and where the carbon is assimilated. The activities of SS increased, which could increase the sugar content in plant leaves, maintain the balance of cell sugar, and maintain the homeostasis and water potential of plant cells. When growth resumed at 25 °C, the sugar content and related enzyme activities of the grafted bitter gourd seedlings were similar to those after heat stress. Therefore, pumpkin and luffa rootstocks are conducive to the rapid recovery of bitter gourd from heat stress.

Among the nitrogen metabolites, ${\text{NO}}_{3}^{-}$ and ${\text{NO}}_{2}^{-}$ contents in heterologously grafted bitter gourd seedlings after heat stress were significantly higher than those in non-grafted and self-grafted seedlings, whereas ${\text{NH}}_{4}^{+}$ showed the opposite trend. Nitrogen metabolism includes nitrogen absorption, transport, amino acid metabolism, and assimilation (Kusano et al., 2011), all of which require energy. However, various environmental stresses lead to insufficient energy metabolism in plants, affecting the synthesis and metabolism of compounds, such as proteins, nucleotides, and chlorophyll (Dai et al., 2014). In watermelon (Yang et al., 2013) and tomato (Sánchez-Rodríguez et al., 2013), grafting promoted nitrogen metabolism in seedlings and improved ${\text{NO}}_{3}^{-}$ assimilation efficiency and NR activity. In our results, under heat stress, grafting promoted nitrogen metabolism, and the ${\text{NH}}_{4}^{+}$ accumulation of luffa rootstock was less than that of pumpkin rootstock. At the same time, compared with non-grafted and self-grafted seedlings, pumpkin and luffa rootstocks increased the activities of NR, NiR, and GS in the leaves of bitter gourd-grafted seedlings after heat stress, and the plants on luffa rootstocks showed better performance. ${\text{NH}}_{4}^{+}$ can not only destroy the photosynthetic system of plants and cause them to experience toxicity but also lead to a reduction in nitrogen utilization efficiency (Skopelitis et al., 2006). The ${\text{NH}}_{4}^{+}$ concentration in plants is not only dependent on NR and NiR catalysis but is also related to ${\text{NH}}_{4}^{+}$ assimilation through the GS/GOGAT pathway. The increased activities of NR, NiR, and GS resulted in a simultaneous increase in ${\text{NH}}_{4}^{+}$ formation and assimilation, effectively reducing ${\text{NH}}_{4}^{+}$ accumulation. In addition, GSH, as a low molecular weight antioxidant and non-protein thiol, plays a key role in regulating intracellular defense by scavenging ROS during stress (Singh et al., 2016), and GSH maintains redox homeostasis as an integral part of the AsA-GSH cycle, so this may also indirectly inhibit the accumulation of ${\text{NH}}_{4}^{+}$ , which needs to be proved by subsequent studies. The increase in enzyme activities related to nitrogen metabolism in heterologously grafted seedlings may be related to the promotion of photosynthesis and antioxidant defense systems of bitter gourd seedlings by grafting, as excessive accumulation of ROS may change the biosynthesis and activity of enzymes (Taïbi et al., 2016). The effects of nitrogen metabolites and enzyme activities under heat stress indicate that strong rootstocks can absorb more nitrogen and promote nitrogen assimilation in leaves. Grafting is also conducive to the recovery of seedlings by inhibiting the accumulation of ${\text{NH}}_{4}^{+}$ . In future research, more attention should be paid to the molecular mechanism of grafting on carbon and nitrogen metabolism of bitter gourd and the improvement of stress resistance of bitter gourd by grafting.

## 5 Conclusions

We found that grafting bitter gourd onto the roots of pumpkin and luffa can help to reduce heat-induced oxidative stress by increasing the scavenging activity of active oxygen, the carbon and nitrogen metabolite content, and the enzyme activity. The possible mechanism is that grafting is conducive to the metabolism of sucrose in bitter gourd leaves, thus promoting the accumulation of non-reducing sugar and reducing sugar, maintaining the balance of cell sugar, the stable state of cells and water potential, and also providing the ability to absorb nitrogen in leaves to prevent cells from being poisoned by ${\text{NH}}_{4}^{+}$ . The grafted seedlings of bitter gourd were subjected to heat stress recovery at 25 °C. We found that pumpkin and luffa rootstocks could quickly restore the normal growth of seedlings. At 40 °C and 25 °C, the performance of the luffa rootstock was better than that of the pumpkin rootstock. Our research shows that the root system of the luffa has a large effect on the adaptability of the scions to heat stress, and this mechanism should be further explored to improve the stress resistance and productivity of plants.

## Data availability statement

The original contributions presented in the study are included in the article/**Supplementary Materials**. Further inquiries can be directed to the corresponding authors.

## Author contributions

LL, HL and YT conceived and designed the experiments. LT designed the experiment and provided grafting technology and guidance. LL, WT, HL and XL performed the experiments. LL, WT and GS analyzed the data. LL and WT wrote the manuscript. LL, BS, ZH, HL and YT reviewed and revised the manuscript. All authors have read and approved the final version of the manuscript. All authors contributed to the article and approved the submitted version.
