# Supplementary material for: Grafting promoted antioxidant capacity and carbon and nitrogen metabolism of bitter gourd seedlings under heat stress
Source: Front Plant Sci. 2022 Dec 15;13:1074889. doi: 10.3389/fpls.2022.1074889 (PMC9798118; doi:10.3389/fpls.2022.1074889)
Supplement: Supplementary file 1 [file DataSheet_1.pdf]

Supplementary Material

1 Supplementary Tables

Supplement Table 1 Importance level of each indicator in PCA

Indicators	PC1	PC2
SOD	0.048	-0.073
POD	0.058	-0.017
CAT	0.061	0.035
Relative conductivity	-0.037	0.163
Protein	0.062	0.026
MDA	-0.055	0.092
Pro	0.054	0.109
$O_2^{\bullet -}$	-0.060	0.058
Soluble sugar	0.059	0.041
Sucrose	0.060	0.060
Fructose	0.057	-0.038
Glucose	0.062	0.038
SPS	0.038	0.145
SS	0.048	0.129
NI	0.060	0.082
AI	0.042	-0.118
NO_3^-	0.027	0.182
NO_2^-	0.056	0.092
NH_4^+	-0.062	0.049
NR	0.054	0.018
NiR	0.048	-0.126
GS	0.056	-0.085
H_2O_2	-0.052	0.105
